# CSF Concentrations of CXCL13 and sCD27 Before and After Autologous Hematopoietic Stem Cell Transplantation for Multiple Sclerosis

**DOI:** 10.1212/NXI.0000000000200135

**Published:** 2023-06-13

**Authors:** Katarina Lundblad, Christina Zjukovskaja, Anders Larsson, Honar Cherif, Kim Kultima, Joachim Burman

**Affiliations:** From the Department of Medical Sciences, Neurology, Uppsala University, Sweden.

## Abstract

**Background and Objectives:**

In the past decade, autologous hematopoietic stem cell transplantation (AHSCT) has emerged as a treatment for relapsing-remitting multiple sclerosis (RRMS). How this procedure affects biomarkers of B- and T-cell activation is currently unknown. The objective of this study was to investigate CXCL13 and sCD27 concentrations in CSF before and after AHSCT.

**Methods:**

This prospective cohort study was conducted at a specialized MS clinic in a university hospital. Patients with a diagnosis of RRMS, treated with AHSCT between January 1, 2011, and December 31, 2018, were evaluated for participation. Patients were included if CSF samples from baseline plus at least 1 follow-up were available on June 30, 2020. A control group of volunteers without neurologic disease was included as a reference. CSF concentrations of CXCL13 and sCD27 were measured with ELISA.

**Results:**

The study comprised 29 women and 16 men with RRMS, aged 19–46 years at baseline, and 15 women and 17 men, aged 18–48 years, in the control group. At baseline, patients had higher CXCL13 and sCD27 concentrations than controls, with a median (IQR) of 4 (4–19) vs 4 (4–4) pg/mL (*p* < 0.0001) for CXCL13 and 352 (118–530) vs 63 (63–63) pg/mL (*p* < 0.0001) for sCD27. After AHSCT, the CSF concentrations of CXCL13 were considerably lower at the first follow-up at 1 year than at baseline, with a median (IQR) of 4 (4–4) vs 4 (4–19) pg/mL (*p* < 0.0001), and then stable throughout follow-up. The CSF concentrations of sCD27 were also lower at 1 year than at baseline, with a median (IQR) of 143 (63–269) vs 354 (114–536) pg/mL (*p* < 0.0001). Thereafter, sCD27 concentrations continued to decrease and were lower at 2 years than at 1 year, with a median (IQR) of 120 (63–231) vs 183 (63–290) pg/mL (*p* = 0.017).

**Discussion:**

After AHSCT for RRMS, CSF concentrations of CXCL13 were rapidly normalized, whereas sCD27 decreased gradually over the course of 2 years. Thereafter, the concentrations remained stable throughout follow-up, indicating that AHSCT induced long-lasting biological changes.

In the past decade, autologous hematopoietic stem cell transplantation (AHSCT) has become recognized as a treatment option for severe relapsing-remitting multiple sclerosis (RRMS).^1^ Like approved disease-modifying drugs (DMDs) for MS, it targets the immune system. Unlike most DMDs, it is a one-time procedure aiming to remove the aberrant self-reactive immune system and to reconstitute a novel immune system.^2^ Studies of AHSCT have repeatedly yielded results that compare favorably with conventional DMDs,^3-5^ and many patients experience long-term remission. Whether biomarkers of inflammation are affected by treatment with AHSCT, and whether such changes are associated with clinical outcome, is hitherto unknown.

Chemokine (C-X-C motif) ligand 13 (CXCL13) is a B-cell attractant and promising biomarker for inflammatory diseases. It is constitutively expressed in lymphoid organs and controls the recruitment of lymphocytes and antigen-presenting cells to these structures. Beyond its role in the development and maintenance of lymphoid tissues, CXCL13 also drives nonlymphoid tissue inflammation. In MS, CXCL13 has been shown to be upregulated in active lesions^6^ but also in tertiary lymphoid structures in the meninges.^7^ It is established as a biomarker of neuroborreliosis^8^ but has also been investigated as a biomarker of MS on several occasions. It is increased in the CSF of patients with active MS^9^ and has been linked to an increased risk of conversion from clinically isolated syndrome to MS^10^ and worse outcome in established MS.^11^

CD27 is a member of the superfamily of tumor necrosis factor receptors and is expressed as a costimulatory molecule on lymphocytes. The interaction between CD27 and its ligand CD70 facilitates the survival and expansion of CD4^+^ and CD8^+^ T lymphocytes and leads to the terminal differentiation of B cells.^12^ sCD27, a 32-kDa protein identical to the extracellular domain of membrane-bound CD27, can be released after lymphocyte activation through shedding from the cell surface by metalloproteinases.^13,14^ The function of sCD27 has not been well characterized, but it has been reported to augment T-cell activation^15^ and induce IgG production in memory B cells.^16^ sCD27 was investigated as a potential biomarker of inflammation in MS already in the 1990s,^17^ with renewed attention recently.^18-20^ CSF concentrations of sCD27 are increased in RRMS and in progressive MS,^18,19^ and it has been linked to an increased risk of conversion from clinically isolated syndrome to MS.^20^

The purpose of this study was to investigate whether intervention with AHSCT for RRMS is associated with a change in the CSF concentrations of CXCL13 and sCD27 in a cohort of patients with RRMS.

## Methods

### Standard Protocol Approvals, Registrations, and Patient Consents

The study was approved by the Regional Ethical Review Board in Uppsala (Dnr 2010/450/1 and 2012/080/1). The study was performed in concordance with the Declaration of Helsinki (1964), and all patients provided written informed consent.

### Patients

The main cohort consisted of 45 patients diagnosed with RRMS according to the revised McDonald criteria^21^ and treated with AHSCT at Uppsala University Hospital in the time period January 1, 2011–December 31, 2018. In total, 85 patients were screened. Patients were included if CSF samples from baseline and at least 1 follow-up were available on June 30, 2020. A control group consisting of 32 volunteers without neurologic disease was included as a reference. Table 1 provides a demographic summary of all patients within the study.

**Table 1:**
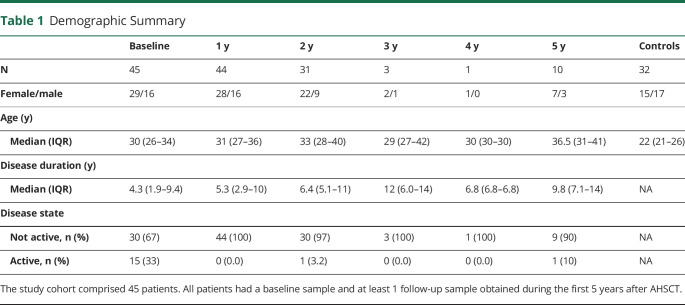
Demographic Summary

Most patients had been treated with DMDs at some time point before AHSCT, and only 5 patients were DMD naive. At baseline, 10 were not currently receiving DMD treatment, 14 were treated with orals and/or injectables, and 21 with natalizumab or rituximab (Table 2).

**Table 2:**
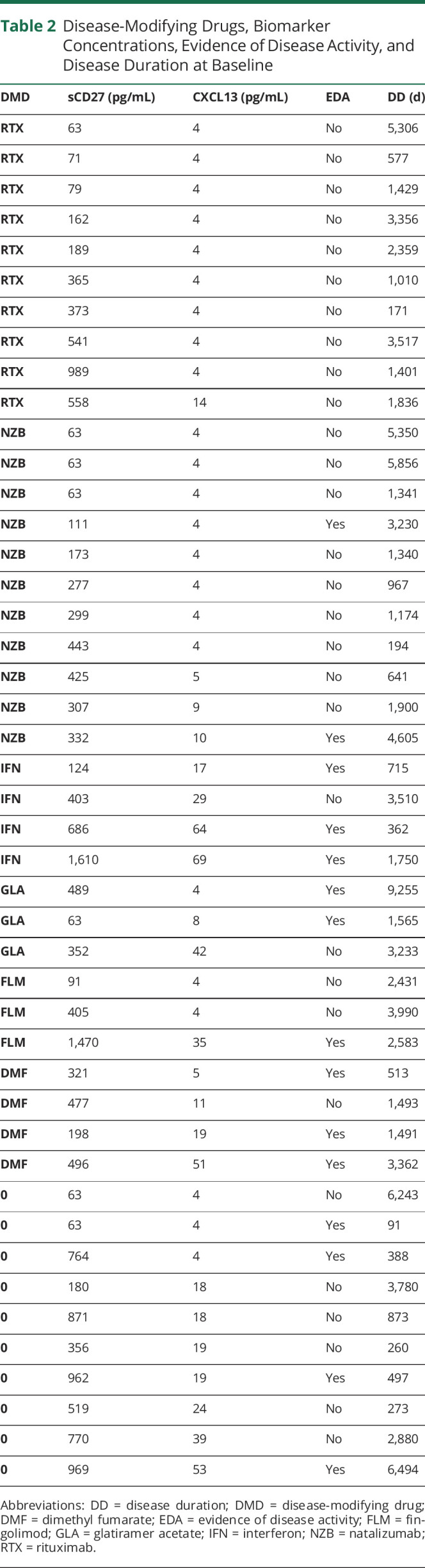
Disease-Modifying Drugs, Biomarker Concentrations, Evidence of Disease Activity, and Disease Duration at Baseline

### Procedures

Autologous hematopoietic stem cells were mobilized with cyclophosphamide and filgrastim as described previously.^5^ No ex vivo graft manipulation was performed. Patients were conditioned with a combination of cyclophosphamide and rabbit antithymocyte globulin (cyclophosphamide 200 mg/kg; rATG 6 mg/kg). Microbial prophylaxis was given as described previously.^5^

### Lumbar Punctures

Lumbar punctures were made as part of health care: before AHSCT and then routinely at follow-up at 1, 2, and 5 years. In a few cases, follow-up with lumbar puncture was postponed, and in some instances, additional lumbar punctures were made. Patients undergoing lumbar puncture were asked to donate 10 mL of CSF for research purposes. The controls underwent lumbar puncture for research purposes at a single time point.

### CSF Handling and Storage

CSF samples were handled according to a 2009 consensus protocol on CSF biobanking.^22^ The CSF samples were handled uniformly and underwent 2 freeze-thaw cycles for aliquotation.

### Quantification of Cells, Albumin, and IgG Index

The CSF cell counts, plasma/CSF albumin, and the IgG index were analyzed by the routine service at the Department of Clinical Chemistry and Pharmacology, Uppsala University Hospital, Uppsala. The laboratory is accredited according to 15189 by Swedac (Borås, Sweden) and is participating in external quality assurance programs organized by Equalis (Uppsala, Sweden). Cell counts were made through manual microscopy by experienced laboratory technicians. Plasma and CSF albumin were analyzed on a cobas pro instrument (Roche Diagnostics, Mannheim, Germany) using reagents and calibrators from Roche Diagnostics. Plasma and CSF IgG were also analyzed on a cobas pro instrument with reagents and calibrators from Roche Diagnostics.

### Quantification of CXCL13 and sCD27 in CSF

All samples were analyzed in the same research laboratory by technicians who were blinded to clinical data. The CSF concentrations of CXL13 and sCD27 were analyzed with commercial ELISA (R&D systems, Minneapolis, MN; catalogue# DY382-05 and DCX130) according to the manufacturer's instructions. Undiluted samples were run in duplex on each plate. The intra-assay CV (pooled CV for all plates) was 4.5% for CXCL13 and 4.4% for sCD27. The lower limit of quantification (LLoQ) and limit of detection (LoD) were set at half the value of the lowest standard point, i.e., 4 pg/mL for CXCL13 and 63 pg/mL for sCD27. Values at or below the LoD were assigned the value of the LoD for the statistical analysis.

### Definitions

A clinical relapse was defined as a period of acute worsening of neurologic function lasting ≥24 hours and not attributable to an external cause such as increased body temperature or acute infection. Confirmed disability worsening (CDW) was defined as an increase in the Expanded Disability Status Scale (EDSS) score with at least 1 point from baseline, sustained between 2 follow-up visits separated in time by no less than 6 months (1.5 points if the EDSS at baseline was 0; 0.5 points if the baseline EDSS ≥5.5). An MRI event was defined as the appearance of any T2 lesion >3 mm or gadolinium-enhancing lesion in the brain or spinal cord not present on the baseline scan. Active disease was defined as the presence of a clinical relapse or at least 1 gadolinium-enhancing lesion ± 1 month from CSF sampling. No evidence of disease activity (NEDA-3) was defined as the absence of clinical relapses, CDW, and MRI events. Patients who did not maintain NEDA-3 were considered to have evidence of disease activity (EDA). Within this study, dimethyl fumarate, fingolimod, glatiramer acetate, and interferons were considered to be first-line treatments, whereas natalizumab and rituximab were considered to be second-line treatments.

### Statistical Analyses

Statistical analyses were performed in GraphPad Prism 9.2 (GraphPad Software, La Jolla, CA). Medians with interquartile range (IQR) were used to summarize data. Correlations were described with Spearman's r. Independent proportions were compared with the Fisher exact test, and the McNemar test was used to compare paired proportions. In comparisons between 2 groups, the Mann-Whitney test was used for unpaired data and the Wilcoxon test for paired data. For unpaired comparisons between more than 2 groups, the Kruskal-Wallis test was used, followed by the Dunn multiple comparison test. To determine the influence of age and/or sex, multiple linear regression analyses were performed. A 2-tailed *p* value of <0.05 was considered statistically significant.

### Data Availability

Anonymized data are available on reasonable request. Data will be available for all types of analyses. Transfer of data will require a signed data access agreement. Requests can be sent per e-mail to the corresponding author, Joachim Burman, joachim.burman@uu.se.

## Results

Eighty-five patients with a diagnosis of RRMS and treatment with AHSCT were evaluated for participation. Forty-five of them fulfilled the eligibility criterion that CSF samples taken before and at least 1 time after AHSCT must be available. A control group consisting of 32 volunteers without neurologic disease was included as a reference. The final study comprised 29 women and 16 men with RRMS, aged 19–46 years at baseline, and 15 women and 17 men, aged 18–48 years, in the control group. Within the main cohort, a total of 134 CSF samples were analyzed: 45 baseline samples and 89 post-AHSCT samples. Baseline samples were obtained at a median (IQR) of 1 (0–11) day(s) before starting the mobilization of stem cells, i.e., 40 (38–56) days before reinfusion of hematopoietic stem cells. The post-AHSCT samples were taken at 1-year follow-up of 44 patients, 2-year follow-up of 31 patients, 3-year follow-up of 3 patients, 4-year follow-up of 1 patient, and 5-year follow-up of 10 patients. The 32 healthy controls underwent lumbar puncture at a single time point alone.

**Figure 1:**
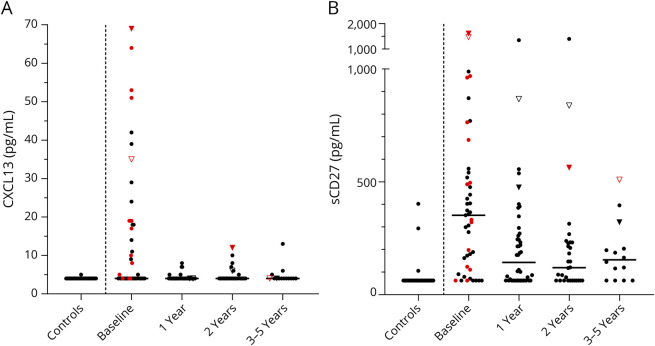
Overview of CXCL13 and sCD27 Concentrations in CSF From Patients and Controls The figure shows an overview of CXCL13 (A) and sCD27 (B) concentrations in CSF of healthy volunteers (n = 32) without neurologic disease and patients with relapsing-remitting MS undergoing autologous hematopoietic stem cell transplantation. A vertical dashed line separates the controls from the patients. Patients were followed with repeated lumbar punctures for up to 5 years after autologous hematopoietic stem cell transplantation (n = 45 at baseline; n = 44 at 1 year; n = 31 at 2 years; n = 3 at 3 years; n = 1 at 4 years; and n = 10 at 5 years). Median values are highlighted by horizontal lines. Active disease (a clinical relapse or gadolinium-enhancing lesions on MRI) at the time when the sample was taken is denoted by red color. Two patients had a clinical relapse in conjunction to a follow-up visit; these patients are represented by triangles (1 filled and 1 open).

We first analyzed whether age and/or sex influenced the biomarker levels. Because neither did, no adjustments for age and/or sex were made. An overview of the unadjusted results is provided in Figure 1. We then assessed the correlations between standard CSF measurements (i.e., monocytes, CSF/serum albumin ratio, and IgG index) and CXCL13/sCD27, excluding values ≤LoD. CXCL13 correlated with monocytes (Spearman r 0.57, *p* = 0.0003), CSF/serum albumin ratio (Spearman r −0.40, *p* = 0.013), and IgG index (Spearman r 0.47, *p* = 0.0036). sCD27 also correlated with monocytes (Spearman r 0.29, *p* = 0.0045) and with IgG index (Spearman r 0.45, *p* < 0.0001), but not with CSF/serum albumin ratio.

### Patients With MS Had Higher CSF Concentrations of CXCL13 and sCD27 Than Healthy Controls

All but one of the healthy volunteers had CXCL13 concentrations below or at the LoD, whereas about half of the patients with MS had detectable concentrations at baseline (1/32 vs 22/45, *p* < 0.0001). Only 4 of the healthy volunteers had quantifiable sCD27 concentrations below or at the LoD, but almost all the patients with MS had detectable concentrations at baseline (4/32 vs 38/45, *p* < 0.0001). Patients with MS had significantly higher concentrations of CXCL13 than healthy controls, with a median (IQR) of 4 (4–19) vs 4 (4–4) pg/mL (*p* < 0.0001). The sCD27 concentrations of patients with MS were also elevated in comparison with controls, with a median of 352 (118–530) vs 63 (63–63) pg/mL (*p* < 0.0001).

### Patients With Active Disease Had Higher CSF Concentrations of CXCL13, but Not sCD27

**Figure 2:**
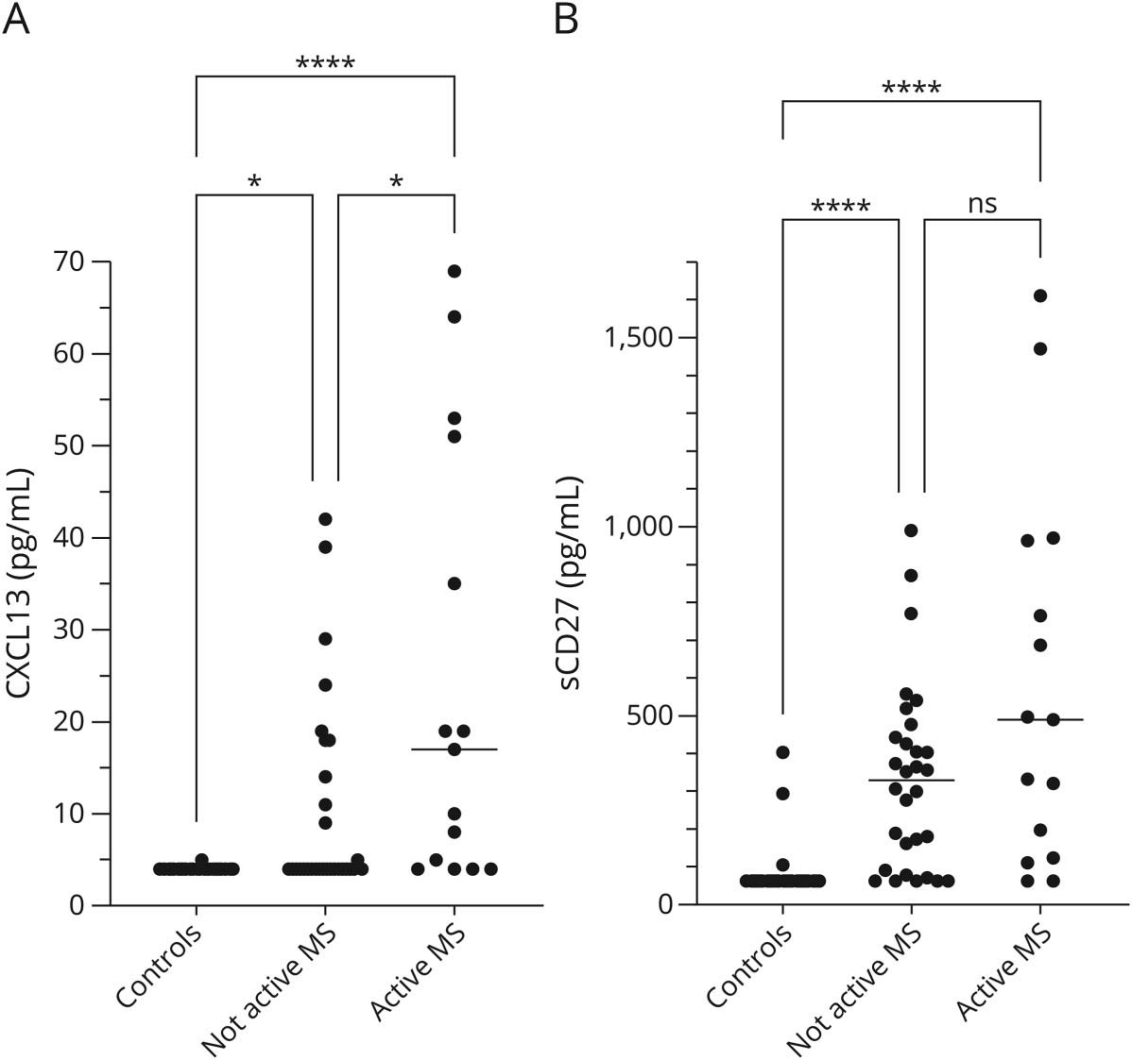
CSF CXCL13 and sCD27 Concentrations by Disease Activity Status CSF concentrations of CXCL13 (A) and sCD27 (B) in healthy volunteers (n = 32) without neurologic disease compared with baseline samples from patients with relapsing-remitting MS (n = 30 not active; 15 active). Statistically significant differences were established with the Kruskal-Wallis test, followed by the Dunn multiple comparison test. CXCL13 was higher in patients with active disease (a clinical relapse or gadolinium-enhancing lesions on MRI) than in controls and in patients with not active MS. By contrast, there was no difference in the CSF concentrations of sCD27 in patients with active or not active disease. Median values are highlighted by horizontal lines. ns = not significant; *p < 0.05; ****p < 0.0001.

To assess whether patients with active disease had significantly higher concentrations of CXCL13 and sCD27 than those without, patients were first separated at baseline by disease status. For each biomarker, a three-way comparison was then made between patients with active/not active disease and healthy controls (Figure 2). Patients with active disease had higher CXCL13 concentrations than patients without, with a median (IQR) of 17 (4–51) pg/mL vs 4 (4–15) pg/mL (*p* = 0.020). However, there was no statistically significant difference in the concentrations of sCD27 between patients with and without active disease: the median (IQR) concentration was 489 (124–962) pg/mL vs 330 (88–452) pg/mL (*p* = 0.94).

### Patients With Second-Line Treatment Had Lower CXCL13 Levels at Baseline

**Figure 3:**
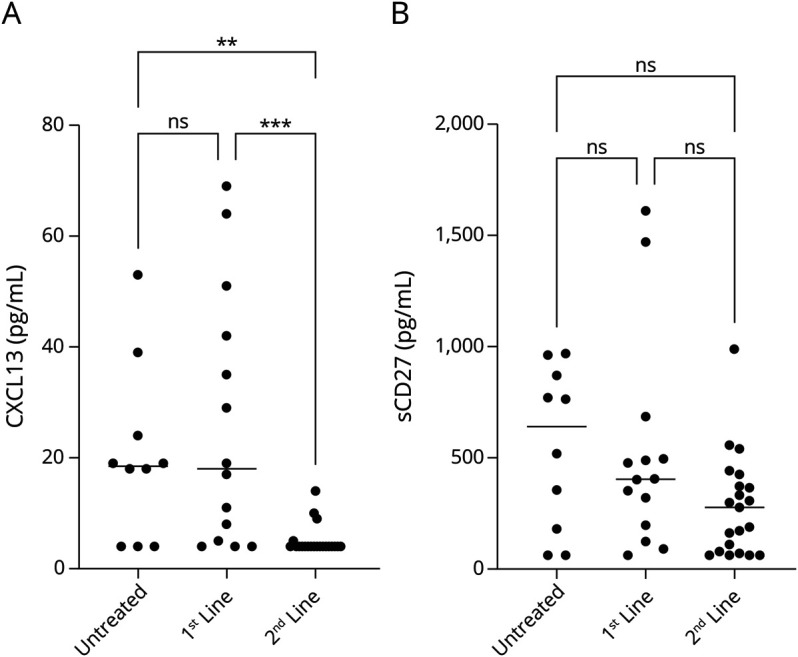
Biomarker Levels and Treatment With Disease-Modifying Drugs Baseline concentrations of CXCL13 (A) and sCD27 (B) in CSF of untreated patients (n = 10) vs patients treated with first-line DMDs (n = 14) vs patients treated with second-line DMDs (n = 21). Statistically significant differences were established with the Kruskal-Wallis test, followed by the Dunn multiple comparison test. ns = not significant.

To assess the influence of treatment with DMDs on the concentrations of CXCL13 and sCD27, patients were separated into 3 groups according to their treatment status at baseline: untreated, first-line treatment, and second-line treatment. Patients with second-line treatment had lower median (IQR) CXCL13 concentration than patients with first-line treatment, 4 (4–4) vs 18 (5–44) pg/mL (*p* = 0.0005), and also lower concentrations than untreated patients, 4 (4–4) vs 19 (4–28) pg/mL (*p* = 0.0077), but there was no statistically significant difference in the concentrations of CXCL13 between untreated patients and patients with first-line treatment, median (IQR) 19 (4–28) vs 18 (5–44) pg/mL (*p* = 1.0). There was no statistically significant difference in the concentrations of sCD27 between any groups: untreated 642 (151–894) pg/mL, first line 277 (180–544) pg/mL, and second line 303 (75–399) pg/mL (*p* = 0.098). See Figure 3.

### Treatment With AHSCT Was Associated With a Rapid Decrease in CXCL13

**Figure 4:**
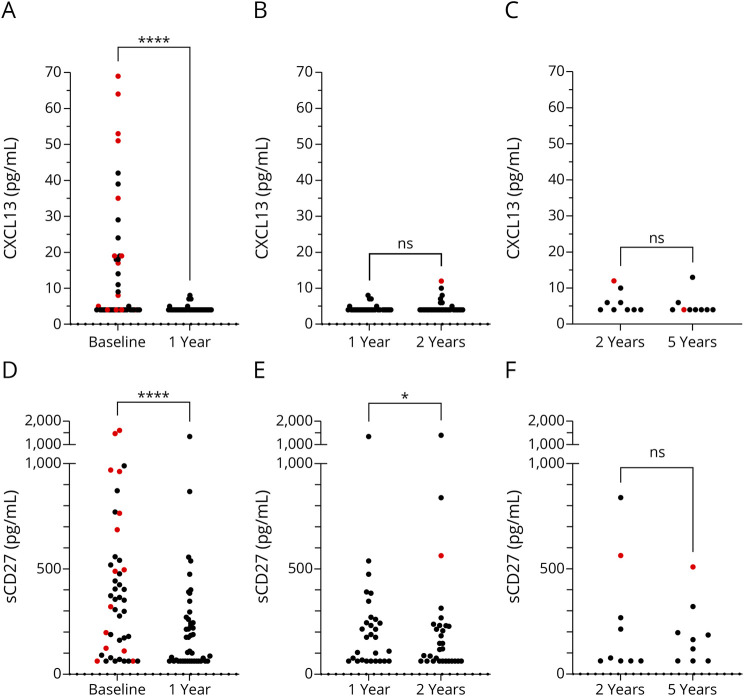
Comparisons of CSF Concentrations of CXCL13 and sCD27 Before and After Treatment With Autologous Hematopoietic Stem Cell Transplantation CSF concentrations of CXCL13 at (A) baseline vs 1 year (44 pairs), (B) 1 year vs 2 years (31 pairs), and (C) 2 years vs 5 years (9 pairs) and CSF of sCD27 at (D) baseline vs 1 year (44 pairs), (E) 1 year vs 2 years (31 pairs), and (F) 2 years vs 5 years (9 pairs). Statistically significant differences were established with the Wilcoxon signed-rank test. Patients with active disease (a clinical relapse or gadolinium-enhancing lesions on MRI) at the time when the sample was taken are denoted by red color. ns = not significant; *p < 0.05; ****p < 0.0001.

Before AHSCT, about half of the patients had CXCL13 concentrations above the LoD, whereas at 1-year follow-up only 5 patients had CXCL13 concentrations above the LoD (21/23 vs 5/39, *p* = 0.001). After AHSCT, the CSF concentrations of CXCL13 were considerably lower at the first follow-up by 1 year than at baseline, with a median (IQR) of 4 (4–4) vs 4 (4–19) pg/mL (*p* < 0.0001). Thereafter, the concentrations of CXCL13 were stable and consistently low throughout the follow-up period (Figure 4, A–C).

### Treatment With AHSCT Was Associated With a Gradual Decrease in sCD27

Before AHSCT, about five-sixths of the patients had sCD27 concentrations above the LoD, whereas at 1-year follow-up about two-thirds had sCD27 concentrations above the LoD (37/7 vs 29/15, *p* = 0.11). After AHSCT, the CSF concentrations of sCD27 were considerably lower at the first follow-up by 1 year than at baseline, with a median (IQR) of 143 (63–269) pg/mL vs 354 (114–536) pg/mL (*p* < 0.0001). Thereafter, the concentrations of sCD27 continued to decrease and were lower at 2 years than at 1 year, with a median of 120 (63–231) vs 183 (63–290) pg/mL (*p* = 0.017). After 2 years, the sCD27 concentrations were stable and consistently low until the end of the follow-up period, with no significant difference between 2-year and 5-year data (Figure 4, D–F).

### Baseline Levels of CXCL13 and sCD27 Were Unrelated to Post-AHSCT EDA

**Figure 5:**
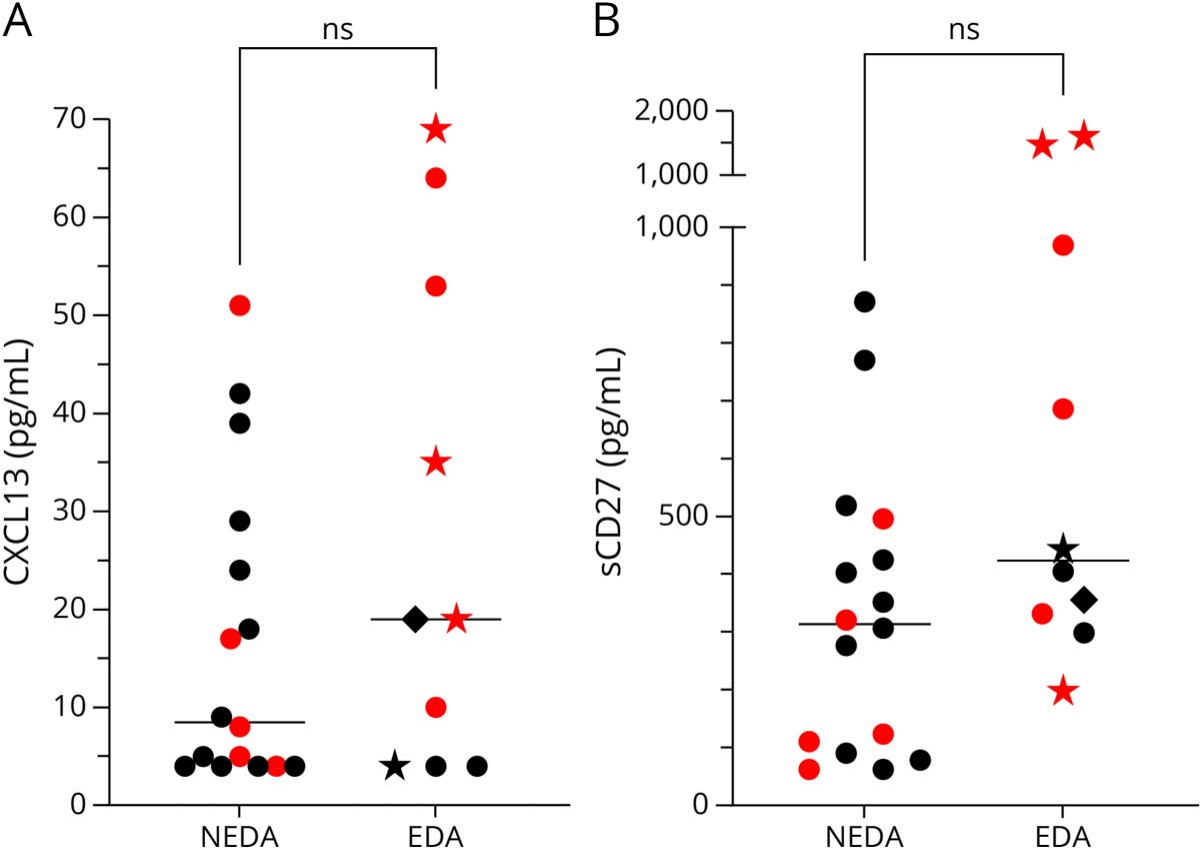
Comparison of Baseline CXCL13 and sCD27 Concentrations in CSF in Patients With and Without Evidence of Disease Activity After Autologous Hematopoietic Stem Cell Transplantation Twenty-six patients treated with autologous hematopoietic stem cell transplantation for MS had follow-up for at least 4 years. Patients with (n = 10) and without (n = 16) evidence of disease activity (clinical relapses, occurrence of new MRI events, or confirmed disability worsening) during follow-up had comparable levels of CXCL13 (A) and sCD27 (B) at baseline. Statistically significant differences were established with the Mann-Whitney test. Median values are highlighted by horizontal lines. Patients with active disease (a clinical relapse or gadolinium-enhancing lesions on MRI) *at baseline* are denoted by red color. Patients with post-AHSCT evidence of disease activity in the form of a clinical relapse are represented by stars, patients with MRI activity alone are represented with dots, and patients with confirmed disability worsening are represented by rhombs. EDA = evidence of disease activity; NEDA = no evidence of disease activity; ns = not significant.

To assess whether the concentrations of CXCL13 and sCD27 at baseline were determinants for EDA during the follow-up period after AHSCT, patients with at least 4 years of follow-up (n = 26) were analyzed further. They were divided into 2 groups based on their NEDA/EDA status. Patients maintaining NEDA-3 throughout the follow-up period had a median (IQR) CXCL13 concentration of 8.5 (4–28) pg/mL; patients with EDA had 19 (4–56) pg/mL (*p* = 0.31). Patients maintaining NEDA-3 throughout the follow-up period had a median sCD27 concentration of 314 (96–478) pg/mL; patients with EDA had 424 (324–1,094) pg/mL (*p* = 0.052). See Figure 5.

### Patients With Post-AHSCT Clinical Relapse

Four patients had a clinical relapse during the follow-up period. Two of them also had active disease at 1 follow-up visit (Figure 1). One was clinically in remission at the time of 2-year follow-up but had active disease on MRI. Sixteen days later, she experienced a clinical relapse, which prompted treatment with rituximab every 6 months for the rest of the follow-up period. The CXCL13 concentrations were 69 – 4 – 12 – 4 pg/mL, and the sCD27 concentrations were 1,610 – 475 – 563 – 321 pg/mL (baseline, 1-year, 2-year, and 5-year follow-up). The increase in CXCL13 and sCD27 coincided with the clinical relapse occurring shortly after the 2-year follow-up. The other had a clinical relapse about 2 months after AHSCT. An MRI scan revealed 1 gadolinium-enhancing lesion in the spinal cord. Subsequent MRI scans did not show activity until the 2-year follow-up, and no additional treatment was instigated. However, 6 months after the 2-year follow-up, the patient had another clinical relapse with a new gadolinium-enhancing lesion in the spinal cord. The patient then started rituximab with infusions every 6 months. Just before the 5-year follow-up, she had another clinical relapse despite treatment with rituximab. This time, the MRI scan showed no activity. The CXCL13 concentrations were 35 – 4 – 6 – 4 pg/mL, and the sCD27 concentrations were 1,470 – 867 – 838 – 509 pg/mL (baseline, 1-year, 2-year, and 5-year follow-up).

## Discussion

A key finding of this cohort study was that the CSF concentrations of CXCL13 and sCD27 decreased after AHSCT, approaching the levels of healthy controls. CXCL13 concentrations decreased rapidly and were normalized within a year, whereas sCD27 concentrations decreased more slowly over the course of 2 years.

In the setting of MS, increased concentrations of CXCL13 in the CSF most likely represent acute inflammation. CXCL13 is undetectable in the normal CNS but upregulated in active MS lesions, whereas not in chronic inactive lesions.^6^ It has also been linked to intrathecal IgG production and accumulation of B and T cells in the CSF of patients with MS.^6,9^ Similarly, it is correlated with the presence of gadolinium-enhancing lesions and clinical relapses.^9,10^ It has also been reported that CXCL13 can be found in ectopic germinal centers in the inflamed meninges of patients with progressive MS.^7^ However, CXCL13 is consistently reported to be lower in progressive forms of MS,^9,11^ and most likely, the contribution of such ectopic germinal centers to the CSF CXCL13 pool is minor. Fitting with this paradigm, we observed that the CSF CXCL13 concentrations correlated with the number of monocytes in CSF and that concentrations of CXCL13 were higher in patients with active disease. CXCL13 has been reported to decrease after treatment with several DMDs.^23^ In this study, patients treated with second-line DMDs had significantly lower levels of CXCL13, in comparison with untreated patients and patients with first-line treatment, accordant with the view that second-line treatment is more effective at suppressing acute inflammation. After AHSCT, CSF concentrations of CXCL13 were consistently low throughout the follow-up.

Like CXCL13, sCD27 is nearly undetectable in the CSF from healthy individuals. It is less clear what the increased CSF concentration of sCD27 stands for in the context of MS. T cells can be induced to produce large amounts of sCD27 in vitro, but other cell types such as B cells and NK cells can also produce sCD27, albeit to a lesser extent.^18^ The cellular source of CSF sCD27 is still unknown, and further histopathologic studies are needed to investigate this. A very real possibility is that sCD27 is produced by multiple sources and reflects activity in multiple pathways. In this study, sCD27 did not show any convincing relationship to disease activity, and it seems to reflect acute inflammation to a much lesser degree than CXCL13. Furthermore, we could not confirm any relationship between sCD27 and the effect of regular DMD treatment, although we observed a tendency toward lower concentrations in patients treated with second-line DMDs. After AHSCT, sCD27 decreased gradually over the course of 2 years, although a considerable portion of patients did not reach normal values at the end of follow-up. Nevertheless, a majority of them had good clinical outcome and maintained NEDA-3 over the observation period. With longer observation time, perhaps some of these patients would reach normal values of sCD27.

In a previous study, CSF CXCL13 was measured before and after AHSCT with an intermediate conditioning regimen.^24^ The follow-up was relatively short (1 year), the number of investigated patients was few, and the patients came from a mixed population of patients with RRMS and patients with progressive disease. Although they used the same assay as in our study, they failed to detect CXCL13 in the samples. The most likely explanation for this is that most of the patients were heavily pretreated with B-cell–depleting agents, such as rituximab and ocrelizumab.^24^ In this study, in which the patients received more various DMDs before AHSCT, patients treated with second-line DMDs had much lower CSF concentrations of CXCL13 at baseline. This reinforces the idea that CXCL13 reflects acute inflammation. In another study, CSF sCD27 was measured before and after AHSCT using a high-intensity conditioning regimen.^25^ The follow-up was relatively short (6–9 months), the number of investigated patients was few, the patients had a secondary progressive disease course, and a different assay was used to measure sCD27. Nevertheless, their results were fairly similar to ours, with a pronounced decrease in the CSF levels of sCD27 in 7 of 8 patients. However, similar to our study, the CSF levels of sCD27 did not reach normal levels.

Clearly, the differential response in CXCL13 and sCD27 suggests that these 2 biomarkers reflect different aspects in the pathophysiology of MS. Both are measurable in serum^17,26^ but at fairly low concentrations, and it is very unlikely that the increased concentrations of CXCL13 and sCD27 in the CSF are a result of an impaired blood-brain barrier. In this study, CSF concentrations of sCD27 were not higher in patients with active disease, but in another study containing a higher number of patients, higher sCD27 CSF concentrations were associated with a small but statistically significant increased risk of new MRI lesions at a follow-up scan of ≤12 months. By contrast, CSF concentrations of CXCL13 were clearly higher in patients with active disease in our study and in a previous study.^10^ The number of gadolinium contrast-enhancing lesions also correlated moderately with CSF concentrations of CXCL13 in another study.^27^ In the context of MS, the cellular source of CSF CXCL13 has convincingly been shown to be infiltrating monocytes/macrophages.^6^ The rapid normalization of CSF CXCL13 after AHSCT indicates that this process has halted. The main source of sCD27 is believed to be activated T cells, and perhaps the decrease in sCD27 seen after AHSCT reflects an inhibition of T-cell activity. In difference to CXCL13, the concentrations of sCD27 were not normalized, and many patients had increased levels at 1-year follow-up. Thereafter, the concentrations of sCD27 continued to decrease over time, similar to what we have observed previously for the IgG index and the presence of oligoclonal bands,^28^ suggesting some linked pathways.

Previous studies had suggested that increased CSF concentrations of CXCL13 and sCD27 were associated with an increased risk of converting from clinically isolated syndrome (CIS) to MS.^10,20^ Because most of these patients were untreated, it would be expected that increased levels in these 2 biomarkers of inflammation increase the risk for future inflammatory events. In another study, the number of relapses over a 5-year period in patients with an established diagnosis of MS was related to the CSF concentration of CXCL13.^11^ The authors did not provide data on treatment status of these patients, but presumably they were not left untreated for 5 years. We therefore sought to determine whether increased CSF concentrations of CXCL13 and/or sCD27 were associated with EDA after treatment with AHSCT. We observed a tendency toward slightly higher baseline values of sCD27 in patients who experienced EDA during the follow-up period but no difference in the values of CXCL13. This may be due to a lack of power because only 26 patients were available for this analysis. In support of this view, the 2 patients with the highest CSF concentrations of sCD27 both experienced a clinical relapse. If an association between sCD27 and an increased risk for future relapses can be proven beyond doubt, performing AHSCT with a more intense conditioning regimen in those patients could be considered. On the other hand, AHSCT may induce an amnestic process, after which pre-AHSCT factors do not affect outcome anymore.

One important limitation of this study is the low number of patients included, especially in relation to post-AHSCT EDA. Another limitation is that not all patients underwent lumbar puncture at all time points. The controls were imperfectly matched in age and sex vis-a-vis the patients. Although these variables did not appear to affect the levels of CXCL13 and sCD27, it is still possible that a weak correlation could exist, which we did not have statistical power to detect. We find it unlikely that this would have affected the outcome of the study. Finally, our CXCL13 assay was not able to discriminate between CXCL13 concentrations <4 pg/mL, which may have affected the outcome of the correlation analyses.

After AHSCT, the CSF concentrations of CXCL13 decreased rapidly and were normalized within a year. This provides a strong argument for the proposition that AHSCT shuts down acute inflammation in MS. The concentration of sCD27 decreased gradually over the course of 2 years; the implication of this is still unclear. We could not confirm that higher baseline levels of CXCL13 or sCD27 were associated with worse outcome.

## Glossary

AHSCT: autologous hematopoietic stem cell transplantation
CDW: confirmed disability worsening
CXCL13: chemokine (C-X-C motif) ligand 13
DMDs: disease-modifying drugs
EDA: evidence of disease activity
EDSS: Expanded Disability Status Scale
LLoQ: lower limit of quantification
LoD: limit of detection
NEDA-3: no evidence of disease activity
RRMS: relapsing-remitting multiple sclerosis
